# Dihydroartemisinin suppresses bladder cancer cell invasion and migration by regulating KDM3A and p21

**DOI:** 10.7150/jca.36174

**Published:** 2020-01-01

**Authors:** Tao Wang, Rongtuan Luo, Wei Li, Houyu Yan, Shunqiang Xie, Wen Xiao, Yongfeng Wang, Bin Chen, Peide Bai, Jinchun Xing

**Affiliations:** The Key Laboratory of Urinary Tract Tumors and Calculi, Department of Urology Surgery, The First Affiliated Hospital, School of Medicine, Xiamen University, Xiamen, China 361003.

**Keywords:** Dihydroartemisinin, bladder cancer, proliferation, migration, invasion, apoptosis, KDM3A

## Abstract

Emerging evidences have shown that Dihydroartemisinin (DHA), used in malaria treatment, possess anti-cancer activity. However, the study of its potential functional roles and the anti-cancer mechanisms in bladder cancer is limited. We performed this study to elucidate the influence of DHA in the biological behavior of bladder cancer cells and tried to explore the molecular mechanism. The results of CCK-8 assay showed that DHA significantly inhibited bladder cancer cell 5637, UMUC3 and T24 proliferation and the inhibitory effect is dose- and time- dependent. Further mechanism study showed that DHA performed its function via down-regulating the expression of histone demethylase KDM3A and inducing p21 expression. Moreover, wound healing and transwell migration/invasion assays revealed that DHA inhibited the ability of migration and metastasis in bladder cancer cell line T24. Finally, flow cytometry and colony formation assays demonstrated that DHA significantly promoted apoptosis of T24 cells and suppressed tumorigenesis as expected. Taken together, our study identifies the anti-cancer capacity of DHA in bladder cancer and explores the underlying mechanism.

## Introduction

Bladder cancer (BCa) is one of the most common malignant tumors in urinary system and its incidence is still very high worldwide 1. In 2015, there were about 80,500 new cases of BCa and it caused 32,900 deaths in China 2. Epidemiological studies showed that genetic and environmental factors such as air pollution, occupational exposure, smoking and passive smoking, diet, drug uptake and gender can affect the occurrence and development of bladder cancer 3-10. The progression of BCa includes a series of biological processes, such as cell proliferation, excessive DNA replication, cell apoptosis and cycle dysfunction, angiogenesis, invasion and metastasis 11, 12. Although radiotherapy and chemotherapy for BCa have made some progress, local progression and metastatic BCa are still unsatisfactory.

Dihydroartemisinin (DHA) is the primary active products of artemisinin and its derivatives 13, which has been used for malaria treatment for many years 14. Recent studies have shown that artemisinin and its derivatives such as DHA exhibited anti-tumor activity in many kinds of tumors, such as ovarian cancer, lung cancer, breast cancer, and prostate cancer 15-19. Mechanistically, DHA suppresses cell proliferation, migration and invasion, as well as induces cell apoptosis 20. The results from Yao Y. *et al*. showed that artemisinin and DHA could suppress cancer-associated fibroblasts induced breast cancer growth and metastasis by suppressing TGF-β signaling to inactivate cancer-associated fibroblasts *in vitro* and *in vivo* 21. Li B. *et al*. performed Western blot and Cell Counting Kit-8 (CCK-8) analysis and their results showed that artemisinin and DHA suppressed the cell cycle-related NF-κB-signaling pathway in epithelial ovarian cancer cells 22. However, the study of the effect of DHA on urinary carcinoma is limited. Thus we hypothesized that DHA may also own anticancer effect on BCa. And we systematically evaluated the functional role of DHA in bladder cancer cell T24 and demonstrated that DHA can inhibit cell growth, migration and invasion. Moreover, DHA can also promote the apoptosis of T24 cells.

Lysine demethylase 3A (KDM3A), also known as Jumonji domain containing 1A (JMJD1A) or JHDM2A or TSGA, belongs to a histone demethylase of the JMJD family 23. KDM3A represent as an histone H3 lysine 9 (H3K9) dimethyl and monomethyl (me2/1) demethylase playing key roles in spermatogenesis 24, energy metabolism 25, stem cell regulation 26 and sex determination 27. Initial study showed that KDM3A was up-regulated and serve an important role in the setting of hypoxia 28. Furthermore, published reports regarding KDM3A has shown its effect on proliferation, migration, invasion, progression and prognosis 29-32, which suggested that KDM3A may be an important marker of cancer and potential target for cancer therapy. Thus, we were in great interest of its expression level regulated by DHA. And our results revealed that DHA down-regulated the expression of KDM3A in T24 cell. In addition, we also found that DHA exposure also induced the expression of cell cycle regulator p21 (cyclin-dependent kinase inhibitor), which acts as an inhibitor of cell proliferation 33. All above explored the anti-cancer mechanism of DHA. Taken together, we systematically investigated the anti-cancer property of DHA in bladder carcinoma and revealed the underlying molecular mechanisms.

## Materials and Methods

### Chemicals and reagents

RPMI-1640 (Cat# 11875093) and fetal bovine serum (Cat# 10099-141) were purchased from Gibco (Grand Island, NY, USA). Dihydroartemisinin (DHA) was purchased from Sigma Aldrich (Cat# D7439, St. Louis, MO, USA). Antibodies against KDM3A (Cat# ab106456) was purchased from Abcam (Cambridge, London, UK), anti-p21 (Cat# sc-471) , anti-GAPDH (Cat# sc-25778) and anti-β-Actin (Cat# sc-8432) antibodies were purchased from Santa Cruz Biotechnology (CA, USA). Protease inhibitor cocktail was purchased from KeyGen (Cat# KGP603, Jiangsu, China). BCA protein assay kit and enhanced chemiluminescent substrate kit were obtained from Pierce (Cat# 23225, Thermo Scientific, Rockford, IL, U.S.A). Cell Counting Kit-8 (CCK-8) was purchased from Med Chem Express (Cat# HY-K0301, Shanghai, China).

### Cell culture

Human 5637, UMUC3 and T24 bladder cancer cells as well as human SV-HUC-1 uroepithelial cells were obtained from the ATCC (Manassas, VA, USA), and the cells were maintained in RPMI-1640 medium (Cat# 11875093, Gibco, Grand island, NY, USA) supplemented with 10% fetal bovine serum (Cat# 10099-141, Gibco, Grand island, NY, USA) and incubated in a humidified incubator at 37 ºC in 5% CO_2_. For DHA treatment, cells were exposed to 25 - 400 μM DHA and harvested at specified time points as indicated.

### Real-time quantitative PCR (qPCR) analyses

For qPCR analyses of mRNA, T24 cells were collected and the reverse transcription was performed with TRIzol (Cat# 15596-018, Invitrogen, Carlsbad, CA, USA)-extracted total RNAs using a PrimeScript™ RT reagent Kit with gDNA Eraser as instructed (Cat# RR047A, TAKARA, Dalian, China). qPCR was performed using the SYBR Green Real-Time PCR Master Mix (Cat# Q-711, Vazyme Biotech Co.,Ltd, Nanjing, China) and the CFX-96 Real-Time PCR system (BIO-RAD, CA, USA) according to the manufacturers' protocols. The primer pairs (Sangon Biotech, Shanghai, China) were used as follows: *Kdm3a*: 5′-GCAAAGGACACGGAGAAGAT-3′ (forward) and 5′-CCCAGCCTTGAACTCCATAC-3′ (reverse); *p21*: 5′-CTGGAGACTCTCAGGGTCGAA-3′(forward) and 5′-TTCCAGGACTGCAGGCTTCCT-3′ (reverse); 18S rRNA: 5′-CGACGACCCATTCGAACGTCT-3′(forward) and 5′-CTCTCCGGAATCGAACCCTGA-3′ (reverse). 18S rRNA was represent as a control and the mRNA levels were normalized using 2^-ΔΔCT^ methods.

### Western blot analyses

T24 cells were lysed by adding RIPA buffer (Cat# R0020, Solarbio, Beijing China). The protein concentration was measured using a BCA Protein Assay Kit (Cat# 23225, Thermo Scientific, IL, USA) and proteins were separated in 12% SDS-polyacrylamide gels and electrophoretically transferred to PVDF membranes (Cat# 10600023, Amersham, CT, USA). After blocked with 5% BSA (Cat# SW3015, Solarbio), the membranes incubated with the primary antibodies, and subsequently incubated with horseradish peroxidase-conjugated secondary antibody. The detection was achieved using the Immobilon Western Chemiluminescent HRP Substrate Kit (Cat# WBKLS0500, Millipore, Darmstadt, Germany). β-Actin or GAPDH levels was analyzed as a control.

### Cell proliferation assay

Cell viability was determined by CCK-8 assay. Human 5637, UMUC3, T24 and SV-HUC-1 cells were seeded in 96 well culture plates (2000 cells/well). After starvation with FBS free medium for 12h, cells were treated with or without 50 - 400 μM DHA for 24 to 72 hours as indicated. Subsequently, 10 µl of CCK-8 reagent (Cat# HY-K0301, Med Chem Express, NJ, USA) was added to each well and allowed for incubation for 4 h. Cell viability was caculated by measuring the absorbance at 450 nm.

### Wound-healing assay

About 5×10^5^ T24 cells were seeded onto 6-well plates. After starvation with FBS free medium for 12h, a wound was incised in the center of the confluent culture, followed by careful washing to remove detached cells and the addition of fresh medium. Subsequently, cells were treated with or without 50 or 100 μM of DHA for 48 hours as indicated. Phase contrast images of the wounded area were recorded using an inverted microscope at indicated time points. Wound area measurement was performed by digital planimetry using Image J software version. The relative wound healing rates were cacalated as the ratio between the healing area at the indicated time point and the original wound area × 100%.

### Migration and Invasion assay

Cell migration was analyzed by Transwell (Cat# 3422, Corning, NY, USA) assays according to the manufacturer's instructions. Matrigel invasion assays were performed using Millicell inserts coated with matrigel (Cat# 354480, BD Biosciences, Sparks, MD, USA). About 2.5×10^4^ T24 cells were seeded per upper chambers in RPMI-1640 containing 1% FBS whereas the lower chambers were loaded with RPMI-1640 containing 5% FBS with or without 200 μM DHA. After 48 hours, the non-migrating cells on the upper chambers were removed by a cotton swab, and migrated cells to the underside of the membrane were fixed with 4% paraformaldehyde and stained with a 0.1% crystal violet solution and counted manually in eight random microscopic fields. The procedure for the cell invasion assays was similar to the cell migration assays, except that the Transwell membranes were precoated with Matrigel (BD Biosciences).

### Apoptosis assay

Annexin V-FITC/PI Apoptosis Detection Kit (Cat# A211-01, Vazyme Biotech Co.,Ltd, Nanjing, China) was used to measure apoptosis. Cells were seeded in 6-well plates at a density of 4 × 10^5^ cells/well and treated with 200 μM of DHA, and control groups were treated with DMSO. Cells were washed with cold PBS after incubated for 48 hours, and then resuspended in Annexin binding buffer, followed by treatment with Annexin V‐FITC and PI reagent for 15 minutes in the dark. Finally, we measured the Apoptosis by flow cytometry.

### Colony formation assay

About 200 of T24 cells were seeded on six-well plates and maintained in RPMI-1640 containing 10% FBS with or without 200 μM of DHA for 2 weeks. The colonies were washed with PBS for twice, and subsequently fixed with 95% ethanol for 15 min and stained with 0.1% crystal violet for 25 min, then slowly washed three times with PBS. When the plate was dried, the number of colonies was counted and photographed. Each experiment was performed in triplicate.

### Other data acquisition, image processing and statistical analyses

Western blot images were captured by Biosense SC8108 Gel Documentation System with GeneScope V1.73 software (Shanghai BioTech, Shanghai, China). Gel images were imported into Photoshop for orientation and cropping. Data are the means ± SEM. The Student's *t* test (two-tailed) for pair-wise comparisons.

## Results

### DHA exposure reduces cell viability in time- and dose-dependent manner in human bladder cancer cells

**Figure 1:**
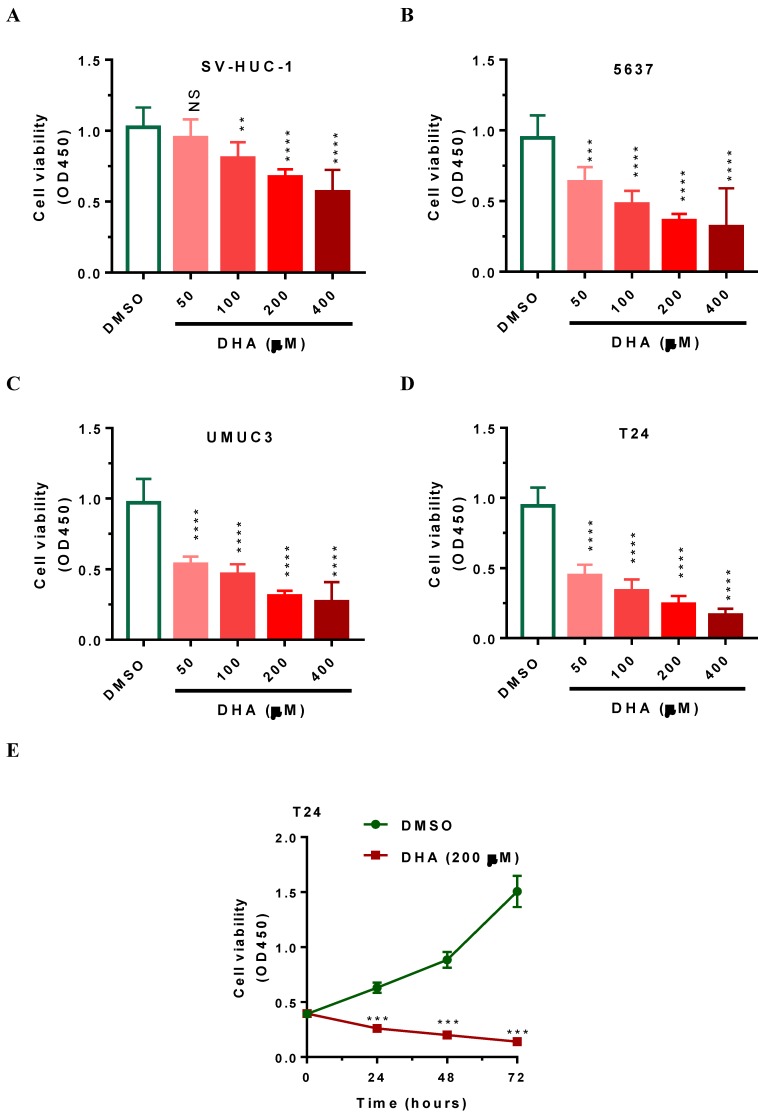
** DHA exposure significantly decreased human bladder cancer cell viability in dose- and time-dependent manner.** Human 5637, UMUC3 and T24 bladder cancer cells as well as human SV-HUC-1 uroepithelial cells cells were seeded in 96-well plate for 24 hours, subsequently, cells were treated with 50 to 400 μM of DHA or DMSO. Cells were then collected at designated time points for CCK-8 analysis. Values represented the means ± SEM for three to four independent experiments. Statistical comparisons were made between the DHA-treated groups versus DMSO-treated groups. NS, not significant; **, *P* < 0.01 ***, *P* < 0.001; ****, *P* < 0.0001. (A-D) DHA exposure significantly decreased SV-HUC-1, 5637, UMUC3 and T24 cell viability in a dose-dependent manner, as determined by CCK-8 assay. (E) DHA exposure significantly decreased T24 cell viability in a time-dependent manner, as determined by CCK-8 assay.

To demonstrate the toxic effects of DHA on cell viability, we subjected human 5637, UMUC3 and T24 bladder cancer cells as well as SV-HUC-1 immortalized uroepithelial cells to DHA at the concentrations from 50 to 400 μM respectively. After 24 hours of treatment, CCK-8 assays were performed to evaluate cell viability. As shown in Figure 1A-1D, DHA treatment significantly reduced the cell viability of bladder cancer cells and SV-HUC-1 uroepithelial cells in a dose-dependent manner. The cell viability was lowest after treatment with 400 μM of DHA for 24 h (Figure 1A-1D). Compared with SV-HUC-1 uroepithelial cells, 5637 UMUC3 and T24 bladder cancer cells are more sensitive in response to DHA exposure (Figure 1A-1D). After treatmen with 50 to 400 μM of DHA, the cell viability (OD450) of T24 was significantly decreased from 0.94 to 0.17 (Figure 1D, P < 0.0001). Furthermore, time course treatment showed that 200μM of DHA suppressed cell proliferation in a time-dependent manner (Figure 1E, *P* < 0.001). Together these data indicated that DHA inhibits the growth of bladder cancer cells and T24 cells are more sensitive to DHA exposure and can serve as a good cellular model for the studies for DHA-induced toxicity.

### DHA down-regulates KDM3A expression and up-regulates p21 expression respectively

**Figure 2:**
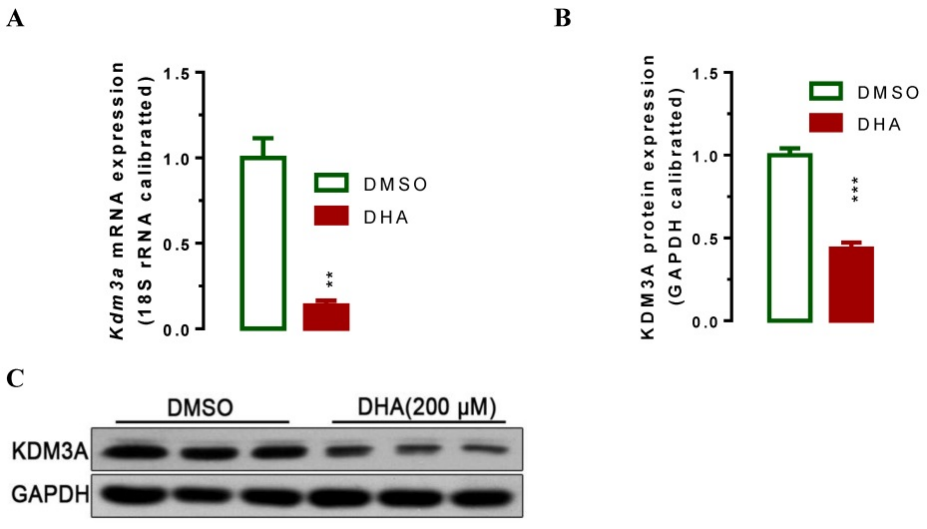
** DHA-induced down-regulation of *Kdm3a* mRNA and protein.** T24 Cells were seeded and allowed to grow for 24 hours to approximately 80% confluency. Subsequently, cells were exposed to 200 μM of DHA. 24 hours after exposure, cells were harvested for mRNA and protein analyses. Values represented the means ± standard error of the mean (SEM) for three independent experiments. 18S ribosomal RNA was used for calibration in real-time RT-PCR analysis of mRNA, and GAPDH served as a loading control for western blotting. **, *P* < 0.01; ***, *P* < 0.001. (A) Treatment of DHA signigicantly decreased *Kdm3a* mRNA expression as determined by RT-qPCR analysis. (B) Optical density scanning showed that DHA significantly suppressed KDM3A protein expression. (C) Western blot analysis demonstrating DHA suppressed KDM3A protein expression.

**Figure 3:**
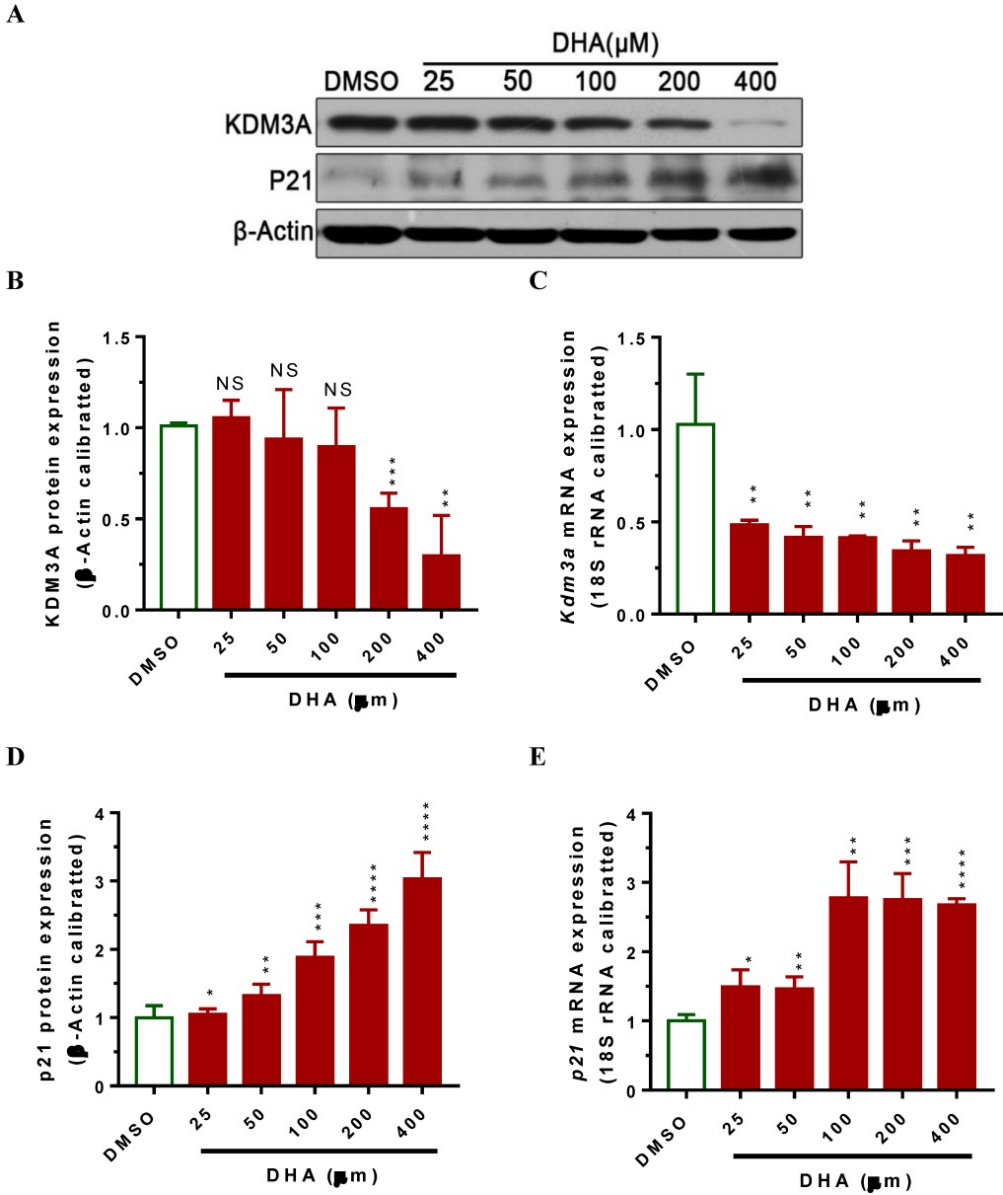
DHA exposure significantly decreased *Kdm3a* mRNA and protein expression, whereas increased *p21* expression in a dose-dependent manner. T24 cells were seeded in 6-well plate and allowed to grow for 24 hours to approximately 80% confluency. Subsequently, cells were exposed to 25 to 400 μM of DHA or DMSO. 24 hours after exposure, cells were collected for mRNA and protein analyses. Values represented the means ± standard error of the mean (SEM) for three independent experiments. 18S ribosomal RNA was used for calibration in real-time RT-PCR analyses of mRNA, and β-Actin served as a loading control for western blotting. NS, not significant; *, *P* < 0.05; **, *P* < 0.01 ***, *P* < 0.001; ****, *P* < 0.0001. (A) DHA-induced dose-dependent regulation of KDM3A and p21 protein in T24 cells. (B) Optical density scanning showed that DHA dose-dependently suppressed KDM3A protein expression. (C) RT-qPCR analysis showed that DHA dose-dependently suppressed *Kdm3a* mRNA expression. (D) Optical density scanning showed that DHA dose-dependently induced p21 protein expression. (E) RT-qPCR analysis showed that DHA dose-dependently induced *p21* mRNA expression.

Lysine demethylase 3A (KDM3A) plays important roles in the metastasis, invasion and development of BCa 34, 35. Cho* et al*. showed that KDM3A was significantly overexpressed in human BCa tissues and knockdown its expression siginficantly suppressed bladder cancer cell proliferation by inducing cell cycle arrest 34. To examine whether DHA exhibited anti-cancer activity via regulating the expression of KDM3A, we used RT-qPCR and western blot assays to determin the KDM3A expression levels in T24 cells after treatment with DHA for 24 h. The results showed that exposure with 200 μM of DHA significantly decreased *Kdm3a* mRNA (Figure 2A, *P* < 0.01) and protein expression (Figure 2B, 2C, *P* < 0.001). Furthermore, treatment with different concentrations of DHA significantly down-regulated KDM3A protein (Figure 3A, 3B) and mRNA (Figure 3C) expression in a dose-dependent manner. By contrast, DHA exposure remarkably up-regulated cell cycle regulation protein cyclin-dependent kinase inhibitor 1 (p21) protein (Figure 3A, 3D) and mRNA (Figure 3E) expression. These results suggest that DHA can regulate bladder cancer cell proliferation by down-regulating KDM3A and up-regulating p21 expression.

### DHA inhibits cell migration and invasion

**Figure 4:**
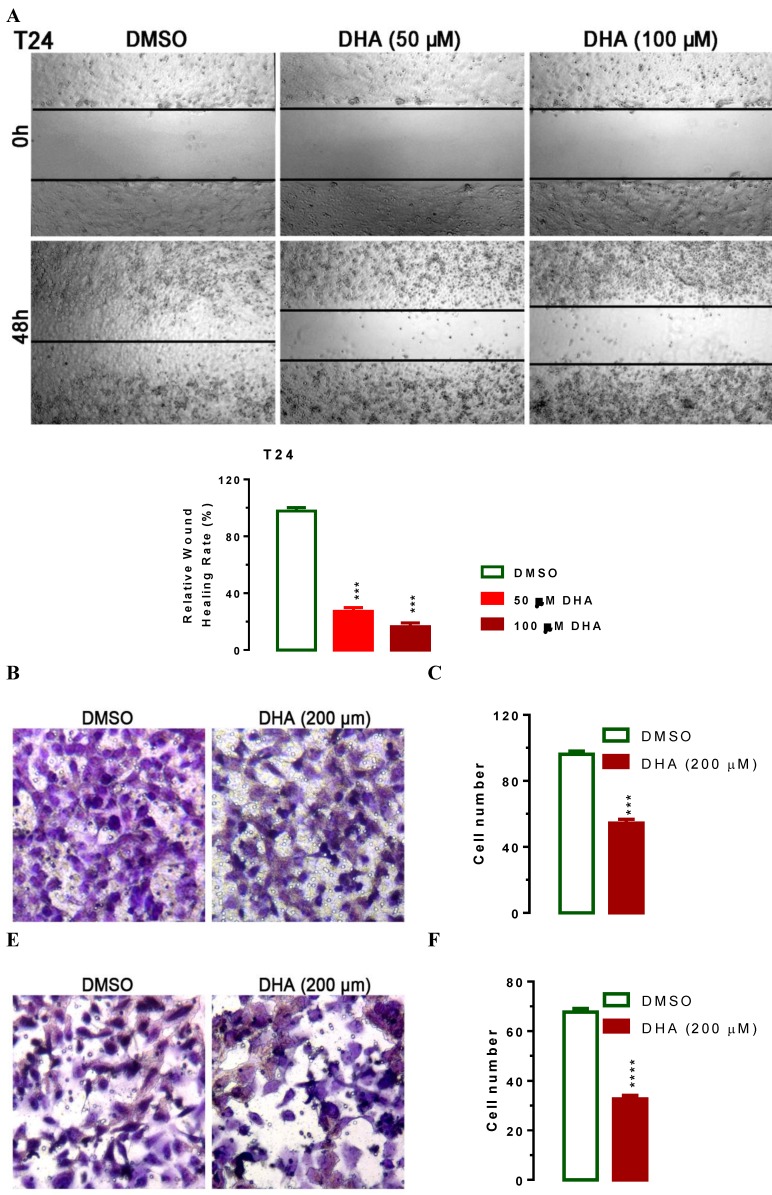
** DHA exposure results in suppressed cell migration and invasion.** For wound‑healing assay, 5×10^5^ T24 cells were seeded onto 6-well plates. After starvation with FBS free medium for 12h, a wound was incised in the center of the plate, followed by careful washing to remove detached cells and the addition of fresh medium. Subsequently, cells were treated with or without 50 or 100 μM of DHA for 48 hours as indicated. Phase contrast images of the wounded area were recorded using an inverted microscope and the relative wound healing rates were caculated at indicated time points. For the migration and invasion assays, about 2.5×10^4^ T24 cells were seeded per upper chambers in RPMI-1640 containing 1% FBS whereas the lower chambers were loaded with RPMI-1640 containing 5% FBS with or without 200 μM DHA. After 48 h, migrated and invaded cells were stained and counted. Data are expressed as the mean ± SEM (*n = 3*). ***, *p* < 0.001; ****, *p* < 0.0001. (A) Treatment of DHA significantly suppressed cell migration as determined in a wound‑healing assay. (B and C) Treatment of DHA significantly suppressed cell migration as determined by transwell migration assay. (D and E)Treatment of DHA significantly suppressed cell invasion as determined by transwell invasion assay.

To evaluate the impact of DHA-mediated regulation of KDM3A and p21, we treated T24 cells with lower concentration of 50 and 100 μM of DHA to exclude the effect of apoptosis on cell migration and performed wound healing experiments. In human T24 cells, DHA exposure dramatically suppressed cell migration in a dose-dependent manner (Figure 4A). Compared with DMSO treated cells, 50 or 100 μM of DHA exposure significant suppressed T24 cell migration (Figure 4A, *P* < 0.001). To further determine whether DHA exposure might affect cell migration and invasion, we performed transwell migration and invasion assays with T24 cells. In the transwell migration assay, we found that DHA treatment significantly decreased the ability of cells to migrate through the membrane by approximately 44% (Figure 4B, 4C, *P* < 0.001) compared with DMSO treatment. Similarly, DHA exposure significantly decreased the likelihood of cell invasion as determined by their ability to penetrate the Matrigel-coated membrane (Figure 4E, 4F,* P* < 0.0001). Together, these data suggest that DHA can suppress Bca cell migration and invasion.

### DHA sensitizes T24 cells to apoptosis and inhibits cell colony formation *in vitro*

**Figure 5:**
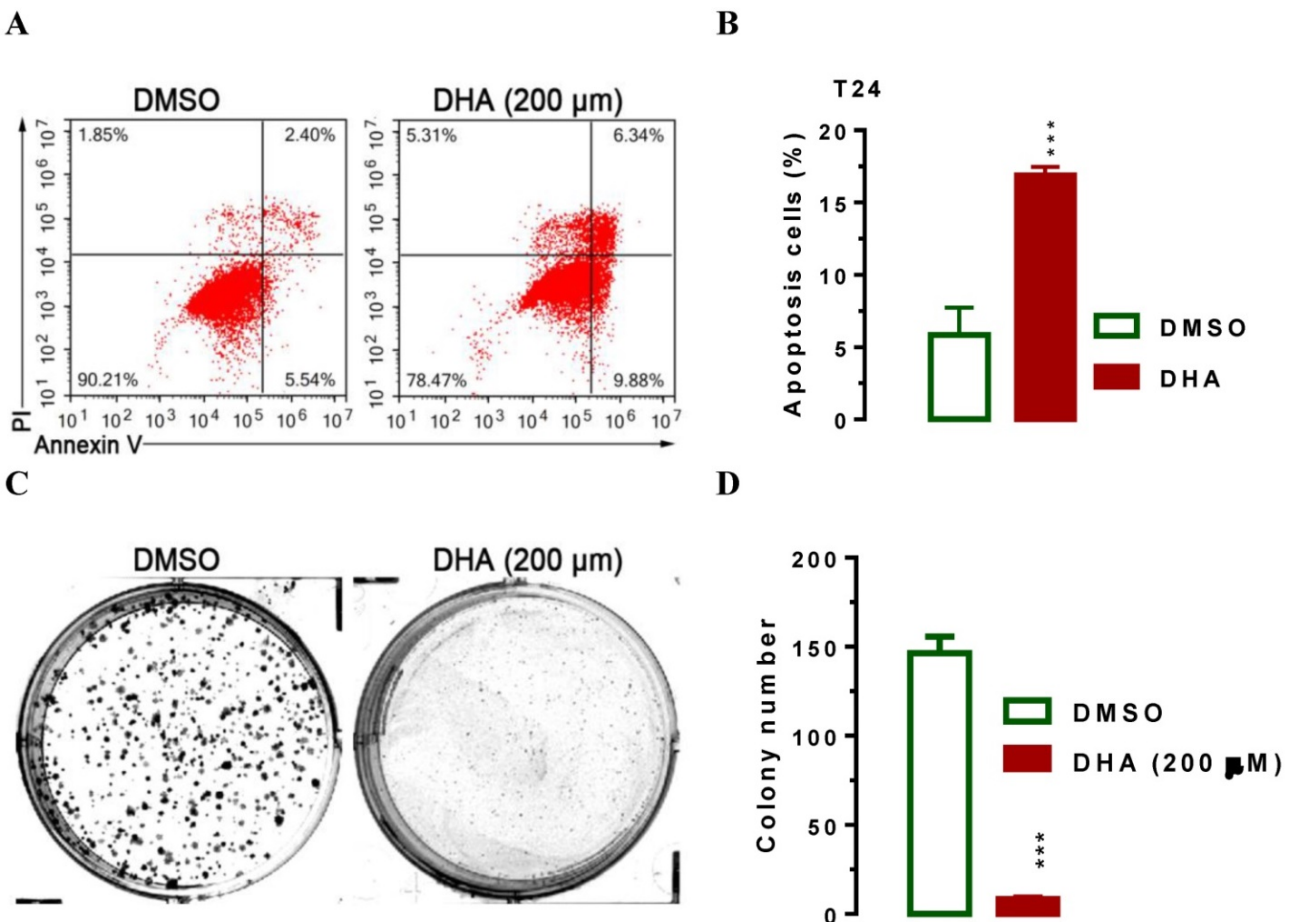
** DHA exposure significantly induced T24 cell apoptosis and suppressed cell colony formation *in vitro*.** For flow cytometry analysis, T24 cells were seeded in triplicate on 6-well plates at the density of ~5×10^5^/well. 24 h after seeding, cells were treated with 200 μM of DHA. 24 hours after the exposure, cells were harvested for analyses as indicated. For colony formation assay, 200 of T24 cells were placed in a fresh six-well plate and maintained in RPMI-1640 containing 10% FBS with or without 200 μM of DHA for 2 wk. Colonies were fixed with methanol and stained with 0.1% crystal violet in 50% methanol for 30 min. Values are expressed as the mean ± SEM. ***, *P* < 0.001. (A and B) Treatment of DHA significantly induced T24 cell apoptosis as determined by flow cytometry analysis. (C and D) DHA exposure significantly suppressed T24 cell colony formation, as determined in a colony fromation assay.

Furthermore, we used flow cytometry assay to investigate the effect of DHA on cell apoptosis. Interestingly, we noticed that the population of cells in apoptosis rose after treatment with 200 μM of DHA for 24 hours (Figure 5A, 5B, *P* < 0.001), indicating that DHA induced cell apoptosis. The significant suppression of migration and invasion by DHA exposure prompted us to further explore the possible biological significance of DHA in tumorigenesis. The capacity for colony formation was evaluated in T24 cells that were treated with 200 μM of DHA or DMSO. Interestingly, DHA treated cells displayed much fewer and smaller colonies compared with DMSO treated cells (Figure 5C, 5D,* P* < 0.001). Together, these results demonstrated that DHA induces BCa cell apoptosis and inhibits cellular tumorigenesis.

## Discussion

As one of the artemisinin derivatives, DHA was used for malaria treatment in the past decades 13, 14. Recent studies showed that DHA represent as a novel and promising drug for cancer therapy exerts anticancer activity in various cancer cells 15-20, 36-38. In this study, we confirm the antitumor effect of DHA on BCa. In human 5637, UMUC3 and T24 bladder cancer cells, DHA-treated cells showed characteristics of inhibited cell proliferation (Figure 1), suppressed cell migration and metastasis (Figure 4) and promoted apoptosis (Figure 5) in dose- and time-dependent manner. A potential therapeutic anti-cancer drug should have high specificity toward cancer cells but not normal cells. To demonstrate the toxic effects of DHA on cell viability, we performed CCK-8 analysis in normal SV-HUC-1 uroepithelial cells and human bladder cancer cells such as 5637, UMUC3 and T24. 24 hours of DHA exposure, 5637 UMUC3 and T24 bladder cancer cells showed more sensitivity in response to DHA compared with SV-HUC-1 uroepithelial cells (Figure 1) suggesting that DHA has high specificity in the treatment of bladder cancer rather than normal cell. Consistent with these features, emerging studies have demonstrated that DHA has little toxic effect on normal cells and was widely used in the treatment of malaria patients 39. All above suggest that DHA should be a novel chemotherapeutic agent against BCa.

The study published by Liu, Y.A.-O.h.o.o., *et al*. reveals that DHA inhibits proliferation, migration and invasion in epithelial ovarian cancer via inhibition of the hedgehog signaling pathway 20. However, the specific molecular mechanism of DHA in BCa is still unknown. In this study, we wanted to explore whether there are any other pathways regulated by DHA in BCa. Based on our previous study, we focused our attention on KDM3A. KDM3A, also called JMJD1A, is a hypoxia-related gene and its expression could be up-regulated in hypoxia 40. Current studies have indentified that KDM3A represent as an oncogene 32, 41 promotes proliferation of hepatocellular cancer cells and confers metastasis and chemoresistance in epithelial ovarian cancer 28, 29. Consistent with these studies, we demonstrated that DHA-treated T24 cells down-regulated the expression of KDM3A in both protein and mRNA levels dose dependently (Figure 2, 3), which resulted in inhibited cell proliferation, suppressed migration, invasion and colony formation, as well as promoted cell apoptosis. Thus, we found a novel signal pathway DHA/KDM3A in BCa.

Studies also showed that KDM3A can regulate cell cycle-related proteins to impact cell cycle process progression 41. Cyclin D1, a cell cycle regulator, was shown to be required for cells to progress through the G1 phase 42. Qin, L., *et al*. have reported that KDM3A enhances proliferation through up-regulating the expression of cyclin D1 41. In our study we found that after treatment with DHA the expression of cyclin-dependent kinase inhibitor (p21) was significantly increased in T24 cells along with the down-regulation of KDM3A (Figure 3), which suggested that KDM3A may also promote proliferation though down-regulating the expression of p21(DHA-KDM3A-p21 pathway). p21 expression has been shown to be regulated largely at the transcriptional level by both p53-dependent and -independent mechanisms 43. Consistent with our results, a recent study demonstrated that KDM3A promoted chemoresistance by demethylating p53 and suppressed pro-apoptotic functions of p53 by erasing p53-K372me1 to impact breast cancer cell invasion and apoptosis 44. These results suggest that KDM3A might suppress p21 expression by regulating p53 under the exposrue of DHA. However, the relative mechenisms need to be further explored. Thus our further work is to confirm this hypothesis using stably KDM3A over-expressive T24 cells.

There are reports showing that DHA induces apoptosis via both intrinsic and extrinsic pathway, respectively through down-regulating the expression of B cell leukemia/lymphoma 2 (Bcl-2) and inactivating NF-κB 36, 37, 45-47. According to these findings, the relationship between KDM3A and Bcl-2, NF-κB as well as other apoptosis- and cell cycle-related genes and the specific regulatoy mechanisms should be elucidated in our further studies.

In conclusion, our study demonstrates that DHA exerts antitumor activity by regulating KDM3A and p21 pathway in BCa. The elucidation of the related mechanism provides theoretical basis for DHA in the treatment of BCa.

## Abbreviations

DHA: Dihydroartemisinin
KDM3A: Lysine demethylase 3A
p21: cyclin-dependent kinase inhibitor
JMJD1A: Jumonji domain containing 1A
Bcl-2: B cell leukemia/lymphoma 2
BCa: Bladder cancer
CCK-8: Cell Counting Kit-8
mRNA: Messenger RNA
DMSO: Dimethyl Sulfoxide
SEM: standard error of the mean
